# Utilizing regression model to characterize the impact of urban green space features on the subjective well-being of older adults

**DOI:** 10.1016/j.heliyon.2024.e35567

**Published:** 2024-08-02

**Authors:** Tianrong Xu, Ainoriza Mohd Aini, Nikmatul Adha Nordin

**Affiliations:** Centre for Sustainable Planning and Real Estate (SUPRE), Faculty of Built Environment, Universiti Malaya, 50603, Kuala Lumpur, Malaysia

**Keywords:** Urban green space, Urban green space features, Old adults, Subjective well-being

## Abstract

Based on the background of rapid global ageing, research exploring urban green spaces and the subjective well-being of urban residents has become one of the key research interests. However, the evidence for assessing the real benefits of urban green spaces on the subjective well-being of older adults by comprehensively examining the features of urban green spaces is limited. We surveyed older urban green space users (n = 536) aged 60 years and older in Nanjing, China, and evaluated the impacts of spatial, green, and grey features of urban green spaces on older people's overall satisfaction with urban green spaces and subjective well-being. The results of the multiple linear regression model showed a strong association between the three types of urban green space features, overall satisfaction with urban green space, and subjective well-being of older adults. The study results indicated that the grey feature of urban green spaces emerged as the most influential factor (p = 0.004) among the three features of urban green spaces in relation to their effect on the subjective well-being of older adults. This implies that it is essential to focus on the importance of grey s of urban green spaces in the planning and design of urban green spaces for older adults' use of different urban green spaces and their sense of well-being. These results contribute to the development of healthy ageing policies and age-friendly urban green space management strategies in rapid ageing countries around the world.

## 1 Introduction

Increased life expectancy is a victory for public health policy and socio-economic development [1,2]. The rapid growth of the ageing population is the most distinctive global trend of the current era [3]. By 2050, the proportion of the world's population aged 65 years and over is projected to rise to 16.00 %, up from 10.00 % in 2022 [4]. The United Nations defines older people as those over the age of 60. The global spread of ageing countries from Europe [5], with several countries experiencing rapid ageing [6], clearly heralds significant changes in the global development dynamics in terms of population size, social groups, economic structure, and regional distribution [7,8]. Based on the context of global ageing, many scholars and policymakers are increasingly focusing on the subjective well-being of older adults as a research topic [9] to help promote the sustainable development of healthy ageing and urban well-being for older adults [10].

Research on subjective well-being has its origins in positive psychology [11], with its philosophic al background in theories of pleasure that emphasize the subjective experience of happiness [12,13], prevalent in fields such as organizational behavior, gerontology, economics, marriage, and epidemiology [14]. Ed Diener first used the concept of subjective well-being in 1984 in his article “*Subjective Well-Being*”. *Subjective well-being* is a convenient and comprehensive psychological indicator of the quality of life of individuals and societies, encompassing people's evaluations of all aspects of life [15]. With the increasing ageing of the global population, numerous studies have attempted to better understand and measure the subjective well-being of older adults from different research perspectives or factors [16,17]. Currently, research on subjective well-being has shown a tendency to become more systematic, in-depth, and applied, with the focus on exploring the factors (e.g., social, economic, personality, demographic, and policy factors) that influence the subjective well-being of older adults [[18], [19], [20], [21]], constructing different theories to explain the psychological mechanisms of the formation of subjective well-being [15,[22], [23], [24], [25]], and developing measurement scales to establish assessment models to express the level of subjective well-being of older adults [[26], [27], [28]].

*Urban green space* is open-space areas reserved for parks and other “green spaces”, including plant life, water features - also referred to as blue spaces - and other kinds of natural environment. and other kinds of natural environment [29]. A qualitative study has demonstrated that exposure to nature and urban green spaces through various pathways can decrease stress and have a positive impact on self-regulation and restorative experiences [30], ultimately resulting in improved health, happiness [31], and well-being [32]. During the Covid-19 pandemic, evidences from epidemiological perspectives also implied that urban green spaces could improve the physical and mental health of the residents [33]. Urban green spaces have become a means of connecting with nature in everyday life [34], with potential links to health and well-being [35]. Urban green space is recognized as a positive sensory and symbolic resource [36,37] that not only fosters a sense of community and social cohesion [38], but through a variety of mechanisms engages in the inevitable experience of well-being [39,40]. Therefore, urban green spaces are one of the most important factors of well-being and quality of life [41] and can positively influence health and well-being in different ways [31]. Research before and during the pandemic proved that people with a greater sense of belonging to the green showed higher levels of well-being [42], and there have been successful studies in countries such as Singapore [43] and Australia [44].

Although existing studies have focused on urban green spaces and well-being, there are some vital research gaps in the current literature that limit our in-depth understanding of this area of research. Firstly, reviews and empirical studies of human-environment interactions in urban green spaces have indicated that most of the existing studies have addressed the target populations of university students [45,46], teachers [47,48], employees [[49], [50], [51]], and doctors [52,53], and lack of studies on the specific group of older adults [54]. Secondly, most of the studies in this field have not examined all the features of urban green spaces in a comprehensive manner, focusing only on a certain category or a few urban green space features, such as the value of ecological services from urban green spaces [55,56], tree canopy and tree richness [57,58], quantity and area of urban parks in the urban built environment [59], quality and safety of recreational and amusement facilities [60], distance to the parks [61], visit frequency [62], and duration of time spent in the parks [63]. Thirdly, previous research has significantly contributed to understanding the relationship between urban green space and subjective well-being in older adults. However, there are notable gaps in the literature regarding the specific types of urban green space features that have the greatest impact on the well-being of older adults. This study aims to address these gaps by exploring the key urban green space factors that influence the subjective well-being of older adults. Additionally, it seeks to offer insights for future landscape planning and design by identifying crucial urban green space features that impact the well-being of older adults. The findings of this study can inform strategies to create urban environments that are more conducive to the well-being of older adults and provide sustainable solutions to the challenges posed by an aging society in the future.

Therefore, our quantitative study, based on a comprehensive examination of all the features of urban green spaces, established the following research objectives: (1). Which features of urban green spaces in the study area impact older adults' subjective well-being? (2). How does overall satisfaction with urban green spaces, obtained by assessing the features of urban green spaces, affect the assessment of subjective well-being of older adults?

Based on this research objectives, we categorized the features of urban green spaces into three types through literature review: spatial, green, and grey features. Subsequently, we formulated three research hypotheses and validated all hypotheses through data analysis to ensure that this study fills existing knowledge gaps and provides insights for future urban green space planning practices. The three hypotheses are as follows.

(1) H1: It was hypothesized that older adults' satisfaction with the spatial, green, and grey features of urban green spaces have a significant impact on their overall green space satisfaction scores.
(2) H2: It is hypothesized that older adults' satisfaction with the spatial, green, and grey features of urban green spaces have a significant impact on their subjective well-being.
(3) H3: It is hypothesized that older adults' overall satisfaction with urban green spaces has a significant impact on their subjective well-being.

This study is outlined as follows: after the introduction, section 2 provides an analytical review of previous literature; section 3 describes the data and methodology; section 4 points out the results of the quantitative measurements; section 5 gives the conclusions and section 6 shows the limitations and future research.

## 2 Methods

This study aims to investigate the influence of older adults’ overall satisfaction with urban green space features on their subjective well-being in post-epidemic Nanjing, China. Questionnaires were designed to achieve this objective. A questionnaire survey was conducted to collect data from people aged 60 years and above. To ensure the accuracy and validity of the collected data, we included validation questions and regulated the response time in the questionnaire. Following data collection, statistical analysis methods were employed to examine the relationship between different features of urban green space and the subjective well-being of older adults.

### 2.1 Literature review

The literature describing the features of urban green spaces is summarized and categorized in a review study that examines in detail the spatial, green, and grey features of urban green spaces [64]. Firstly, spatial features primarily describe a range of properties related to the physical space of the green space [59]. Spatial features such as size, type, shape, location, quantity, quality, distance to parks, availability, safety, accessibility, visit frequency, and duration within the green space through urban green spaces were used to interpret the relationships between them and older people's subjective well-being. Secondly, green features reveal a range of characteristics mainly related to the ecology of green spaces [65,66]. By providing plant habitats [67] and enhancing plant abundance [57], positive contributions can be made to ecosystem services such as climate regulation and air purification [68]. In addition, maintaining carbon and oxygen balance, water resources, reducing urban noise, bird and animal species richness, providing landscape aesthetic space, and socio-cultural-ecological interactions are also included in the green features of urban green spaces to explore their role in promoting subjective well-being levels of older people. Thirdly, grey features refer to the various facilities provided in the urban green space [69,70], which mainly include rest, parking, exercise, sanitation, lighting, security, instruction, landscape, and management facilities. The provision of quality grey facilities is an essential element in attracting visitors to urban green spaces [71] and plays an important role in promoting green space visitation rates [72].

### 2.2 Study area

**Fig. 1:**
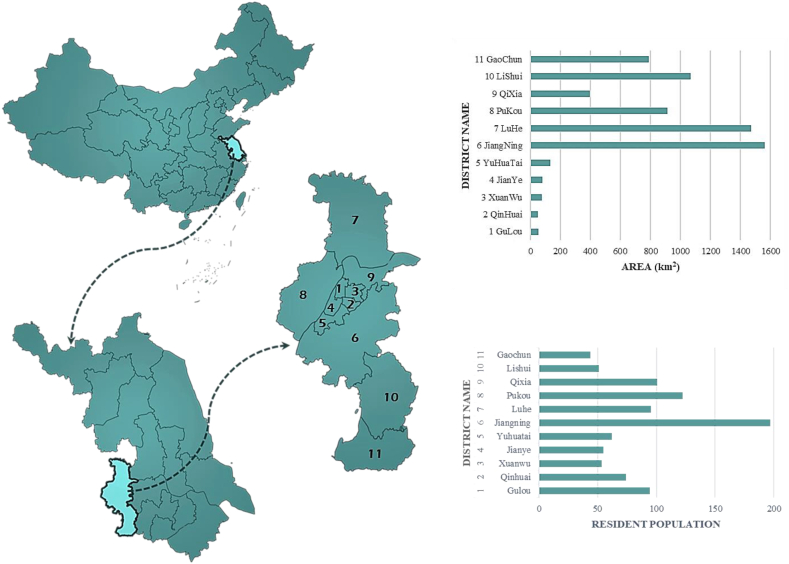
Geographic location of Nanjing, area, and population of districts. (Source from: Statistical Bulletin on National Economic and Social Development, Nanjing Municipal Bureau of Statistics. 2022).

The focus of this study is Nanjing, China, which is located in the eastern part of the country and the central area of the lower reaches of the Yangtze River (between 31°14″ to 32°37″ north latitude and 118°22″ to 119°14″ east longitude). Nanjing is an important center city in eastern China. Nanjing is divided into 11 districts, with a total area of 6587.02 square kilometers. The built-up area covers 868.28 square kilometers. As of the end of 2022, the permanent population of Nanjing has reached 9.49 million people. Jiangning District is the largest in terms of area and has the highest population among all the districts in Nanjing. Fig. 1 shows the geographic location of Nanjing in China, the division of its districts, and the distribution of population.

### 2.3 Target population

**Fig. 2:**
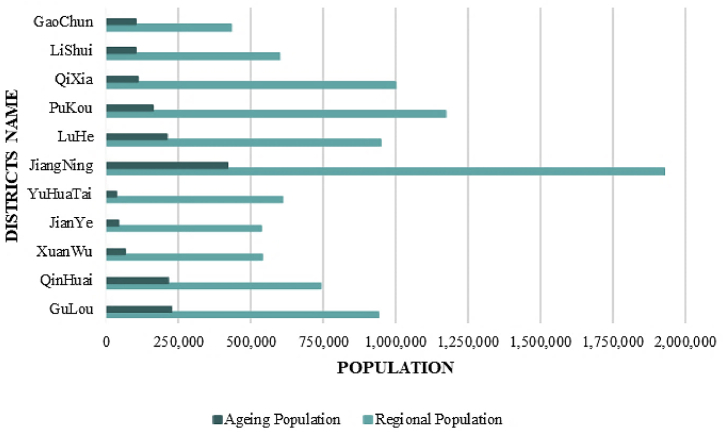
Current Status of Ageing by Districts in Nanjing. (Data from: Nanjing Health Care Commission, 2023).

Nanjing has entered a stage of profound ageing. As of the end of 2022, the population of residents aged 60 and above in Nanjing reached 1.82 million, accounting for 19.36 % of the total population. It is noteworthy that both Gulou District and Jiangning District have exceeded 220,000 older residents, constituting a proportion exceeding 22.00 %. The population aged 65 and above is 1.36 million, accounting for 14.49 %, with Jiangning District being the most prominent, reaching 216,800 people. In contrast, the proportion of older adults’ population in Jianye District and Yuhuatai District is less than 10.00 %. Fig. 2 illustrates the specific ageing status in each district of Nanjing. This data highlights the evident growth of the ageing trend in Nanjing, posing new challenges to the city in terms of social, medical, and older care services.

### 2.4 Inclusion criteria

The target population for this study comprises individuals aged 60 and above in Nanjing city. According to the latest data released by Nanjing city, the number of older individuals with Alzheimer's disease exceeds 100,000. Additionally, approximately 55,000 older individuals have been excluded from this survey due to vision, hearing, and language impairments. After further segmentation of the target population, it is estimated that around 1.70 million older individuals meet the inclusion criteria for this study. Based on the analysis of the target population and the interpretation of the survey objectives, the inclusion criteria for this survey are as follows.

(a) Older adults aged 60 or above;
(b) Older adults who have been living in community areas of various districts in Nanjing rather than in nursing homes or other older care institutions;
(c) Older adults who are physically healthy;
(d) Older adults who are mentally healthy;
(e) Older adults who plan to visit parks rather than just passing through them;
(f) Older adults without cognitive impairment.

These inclusion criteria are prerequisites for accurate sample selection to ensure the representativeness and reliability of the survey results.

### 2.5 Sample size

This study employs the adjusted Yamane formula [64] to determine the optimal sample size for both continuous and categorical variables at all confidence levels. The calculation results indicate that, at a confidence level of 95 % and a margin of error of 0.05, the minimum sample size required is 384 responses.

$$n=\frac{N}{1+N\varepsilon^{2}}$$

Where.

n = minimum returned sample size
N = Population size = 1700000
e = the degree of accuracy expressed as a proportion = 0.05
ρ = the number of standard deviations that would include all possible values in the range = 2
t = t-value for the selected alpha level or confidence level at 95 % = 1.96
ɛ = adjust margin of error [$\varepsilon =\frac{\rho e}{t}$]
→ $ɛ=\frac{2*0.05}{1.96}=0.051$.
→ $n=\frac{1700000}{1+1700000*{\left(0.051\right)}^{2}}=384$.

However, the sample size of the test dataset has a profound impact on the assessment of the model's predictive ability [65]. This means that the larger the test sample size, the higher the estimated accuracy values, and the more favorable it is for interpreting the study. Additionally, Nanjing city has a diverse range of urban green space types, and an increased sample size is beneficial for better model construction and accurate evaluation. Therefore, to articulate this study more accurately, we decided to increase the sample size from 384 to over 500. In the actual survey, a total of 536 valid samples were collected. Overall, a detailed analysis of the background information of 536 respondents was conducted using descriptive statistical methods. Among them, 38.20 % of the respondents fall within the age range of 66–70 years old. The sample is predominantly male (51.30 %), with 56.00 % having a high school education or higher. Urban residents account for 56.30 % of the sample, and the married population constitutes 60.30 %, while the proportion of divorced, unmarried, or widowed individuals is 39.70 %.

Furthermore, 46.30 % of the respondents reported having religious beliefs, while others stated either no religious affiliation or were uncertain. Before retirement, 33.60 % of the respondents worked in the private sector. The highest proportion of respondents with incomes ranging from 6000 to 8000 RMB was 22.90 %. In terms of the distribution of the sample across different districts, Qinhuai District had the highest proportion at 14.00 %, while Yuhuatai District had the lowest at 3.70 %. These sample data align with the current level of ageing in each district, indicating a high level of representativeness.

### 2.6 Sampling

In this study, two sampling methods, stratified sampling and random sampling were employed. The stratified sampling involved dividing the city of Nanjing into 11 administrative districts. Using the ageing ratio as a weight and multiplying it by the total sample size (n = 536), the required sample size for each district was determined. For instance, in Gulou District, with an ageing ratio of 13.14 %, the calculated sample size was approximately 71. To ensure overall representativeness, it was crucial to balance the sample sizes across districts concerning ageing ratios. Table 1 shows the sample size for each district.

**Table 1:** Sample size in each district.

Study Area	Sampling Method	Target Population	Layers	District Name	Sample Size
Nanjing	Stratified Sampling	1,700,000	n1	Gulou	71
			n2	Qinhuai	69
			n3	Xuanwu	23
			n4	Jianye	17
			n5	Yuhuatai	14
			n6	Jiangning	125
			n7	Lvhe	64
			n8	Pukou	50
			n9	Qixia	36
			n10	Lishui	33
			n11	Gaochun	34
				Total	536

Within each district, random sampling was employed to select samples, ensuring that individuals had an equal chance of being chosen. The samples obtained through stratified sampling were used to estimate the overall ageing situation in Nanjing, while those from random sampling within each district were used eco estimate ageing conditions specific to each district. In the overall estimation, results from each district were weighted according to the ageing ratio to better reflect the impact of each district in Nanjing. Therefore, the adoption of a stratified sampling design in this study contributes to improving the efficiency and precision of estimating the ageing situation in Nanjing. Simultaneously, it ensures randomness and representativeness within each geographical area.

### 2.7 Urban green space sampling

Nanjing is renowned as the “Gardens of City.” As of 2021, the coverage rate of greenery in the built-up areas is 44.96 %, and the green space rate is 40.82 %, with a per capita park green space area of 16.20 square meters. These three indicators rank among the top in similar cities in China. According to the Chinese Urban Green Space Classification Standard (CJJ/T85-2017), urban green spaces within construction land can be categorized into five main types, including Park Green Space (G1), Protective Green Space (G2), Square Land (G3), Ancillary Green Space (XG), and Regional Green Space (EG).

In this study, Protective Green Space is excluded from the research scope due to its primary functions related to hygiene, isolation, and ecological protection, making it unsuitable for public access. Square Land, with a green coverage requirement of 65.00 % or more, is only considered Park Green Space. However, due to limitations imposed by this criterion and considering the time frame of the investigation, Square Land is also excluded from the study. Additionally, Ancillary Green Space primarily involves public management, public service facility land, road and transportation facility land, logistics and storage land, industrial land, etc., and is therefore excluded from this research. To ensure coverage of green space types in all 11 districts of Nanjing, this study focuses on Comprehensive Parks, Wetland Parks, Riverside Parks, Community Parks, Urban Forests, and Pocket Parks. These selected green space types aim to reflect the diverse functions and features of urban green spaces within construction land in Nanjing.

To ensure that the surveyed participants meet the criteria within the designated areas, the sampling of urban green spaces utilized a distribution map, incorporating 66 parks into the study (see Supplementary Material 1). The criteria for selecting urban green space sampling points included.

(a) Accessibility of urban green spaces for the public and older adults.
(b) Adequate safety of urban green spaces for older adults.
(c) Consideration of the number of older adults to urban green spaces in the study.

### 2.8 Questionnaire

This study aims to investigate the satisfaction of individuals aged 60 and above in the Nanjing region of China regarding urban green spaces, including spatial, green, and grey features, and to examine how the overall satisfaction with urban green spaces influences their subjective well-being. The research methodology involves a literature review and the design of a questionnaire on urban green space satisfaction. Table 2 outlines the measurement indicators encompassing spatial, green, and grey feature satisfaction, which collectively contribute to the assessment of overall satisfaction among older adults with urban green spaces.

**Table 2:** Urban green space features.

Spatial Features	Green Features	Grey Features
**Shape**	Maintaining carbon and oxygen balance	Car parking facilities
**Area**	Purifying the environment	Resting facilities
**Type**	Improving the urban microclimate	Activity facilities
**Distance**	Disaster prevention and mitigation	Sports facilities
**Quantity**	Biodiversity conservation	Sanitary facilities
**Quality**	Vegetation richness	Lighting facilities
**Availability**	Water resources	Security facilities
**Safety**	Bird and animal species richness	Directional facilities
**Frequency**	Providing aesthetic spaces	Landscape facilities
**Duration**	Social, cultural, and ecological interactions	Park Management centers
		Service facilities buildings
		Path facilities
		Accessibility facilities

On the other hand, the study employs the Well-being of Older People (WOOP) scale to collect self-reported happiness scores from older adults. The WOOP scale, developed by Hackert [66], is a comprehensive tool specifically tailored to evaluate the well-being of older adults. This scale encompasses various domains relevant to older adults' well-being and has been validated through both quantitative and qualitative research [67]. The WOOP scale offers a unique framework that is widely utilized for accurately assessing the well-being of older adults [[67], [68], [69]]. Table 3 shows the indicators included in the WOOP scale to measure the well-being of older adults.

**Table 3:** Indicators in the Well-being of Older People (WOOP) scale.

Indicators Name
Physical health, Mental health, Social life, Receive support, Acceptance and resilience, Feeling useful, Independence, Making ends meet, Living situation

Through these measurement tools, the research aims to comprehensively understand how older adults perceive and evaluate urban green spaces and analyze the relationship between these evaluations and their well-being. The study design intends to uncover the impact of urban green spaces on the quality of life for older adults, providing valuable information for urban planning and the welfare of older adults.

### 2.9 Data collection and analysis

This study employed a Green Space Satisfaction Questionnaire to conduct face-to-face surveys at urban green space sampling points, collecting satisfaction data from older adults regarding the spatial, green, and grey features of urban green spaces. Based on this, an overall satisfaction rating for urban green spaces was completed. Additionally, a Happiness Questionnaire was used to collect self-reported subjective well-being data from older adults. Data collection took place from August 12, 2022, to September 12, 2022.

Before commencing the survey, participants were briefed on the study's objectives and contents. The formal questionnaire was only initiated after the participant had signed a written consent form. To ensure the quality of the questionnaire, respondents were instructed not to exceed 30 min when completing it, preventing participant fatigue due to prolonged questionnaire completion. For participants unable to read paper questionnaires or complete online surveys, interviews were conducted to interpret the questionnaire and assist participants in completing it to ensure valid data. Additionally, this study did not include individuals unwilling to participate in the survey; instead, only valid questionnaire responses were collected.

To explore the impact of green space feature satisfaction and overall green space satisfaction on the subjective happiness of older adults, quantitative techniques were employed. Statistical analysis was performed using SPSS 26.0 software, with a set matching 95 % confidence interval and a statistical significance threshold of 0.05. Correlation data analysis was conducted for all independent and dependent variables included in the study to address each research hypothesis. Moreover, before conducting linear regression analysis, covariance diagnosis is carried out using methods such as variance inflation factor (VIF) and condition number test. The analysis reveals that all tolerance values fall within the range of 0–1, VIF values are below 10, and condition numbers are below 100, indicating the absence of covariance among the predictor variables in the regression model. The results of collinearity diagnosis are presented in supplementary material 2. Finally, by constructing a multiple linear regression equation, the statistical analysis results were interpreted.

## 3 Results

Here, we examined the impact of urban green space spatial, green, and grey features (independent variables) as well as visitation frequency on the subjective well-being of older adults (dependent variable) through the results of multiple linear regression analysis. The outcomes of statistical and content analysis contribute to a more reliable understanding of the relationship between urban green space features and the subjective well-being of older adults.

### 3.1 Descriptive statistics of the visit frequency of urban green space for older adults

Descriptive statistics were used to describe the frequency of respondents' visits to green spaces (as shown in Table 4). The results show that the most reported frequencies were visiting the park once every two weeks (27.20 %); three or more times a week (23.30 %); once or twice a week (22.20 %); once a month (14.40 %); and never (12.90 %).

**Table 4:** Descriptive statistics of the visit frequency of urban green space for older adults.

NO.	Details	Frequency	(%)
**1**	Three times a week and above	125	23.30
**2**	Once or twice a week	119	22.20
**3**	Once every two weeks	146	27.20
**4**	Once a month	77	14.40
**5**	Not at all	69	12.90
	Total	536	100.00

### 3.2 Visit frequency, overall satisfaction with urban green spaces, and the subjective well-being of older adults

The visit frequency to green spaces was utilized as the primary dimension to investigate its correlation with the satisfaction of urban green spaces and the subjective well-being of older adults. As depicted in Table 5, the frequency of visits exhibited a statistically significant correlation with both satisfaction with urban green spaces (p = 0.005) and the subjective well-being of older adults (p = 0.045). The data suggest that the more frequently older adults visit urban green spaces, the higher their satisfaction with these spaces. Conversely, survey respondents indicated that those who frequently explored green spaces were more likely to report higher life satisfaction and exhibit higher levels of positive emotions, along with lower levels of negative emotions, compared to those who never visited green spaces. Consequently, self-reported subjective well-being and satisfaction with green spaces tend to increase with frequent visits to urban green spaces by older adults.

**Table 5:** Visit frequency, overall satisfaction with green spaces, and the subjective well-being of older adults.

Variables	Overall satisfaction with urban green space			Subjective well-being of older adults		
	95 % CI	Pearson Correlation	*p*-value	95 % CI	Pearson Correlation	*p*-value
**Visit Frequency**	−0.507 ∼ −0.184	0.121^a^	0.005	−0.866–−.548	0.086^b^	0.045

a Significant at 1% level.

b Significant at 5% level.

### 3.3 Satisfaction with urban green space features

Table 6 presents the respondents' satisfaction ratings regarding the spatial, green, and grey features of urban green spaces in Nanjing. Over 62.00 % of the respondents rated their satisfaction with these three main features within the range of 3–5. Among them, essential satisfaction (3 points) saw green features (32.80 %, n = 176) accounting for the highest percentage, followed by grey features (31.90 %, n = 171), with spatial features being the lowest (25.00 %, n = 134). For those who were satisfied (4 points), spatial features received the highest percentage (26.30 %, n = 141). In the case of very satisfied (5 points), green features took the lead with the highest percentage (13.60 %, n = 73), while spatial features (11.20 %, n = 60) and grey features (11.00 %, n = 59) were closely ranked. Furthermore, respondents expressed the most significant dissatisfaction with the spatial features of urban green spaces (37.50 %, n = 201), with 18.80 % (n = 101) indicating dissatisfaction and 18.70 % (n = 100) indicating very dissatisfaction. The grey feature had the highest dissatisfaction score (20.00 %, n = 107) but the lowest very dissatisfaction score (12.90 %, n = 69). The statistics also indicated that urban green spaces received the highest satisfaction scores for green features (67.50 %, n = 362).

**Table 6:** Descriptive statistics of satisfaction with urban green space features.

NO.	Details	Satisfaction with the spatial features		Satisfaction with the green features		Satisfaction with the grey features	
		Frequency	(%)	Frequency	(%)	Frequency	(%)
1	Very Unsatisfied	100	18.70	76	14.2	69	12.90
2	Not satisfied	101	18.80	85	15.9	107	20.00
3	Generally satisfied	134	25.00	176	32.8	171	31.90
4	satisfied	141	26.30	126	23.5	130	24.30
5	Very satisfied	60	11.20	73	13.6	59	11.00
6	Total	536	100.00	536	100	536	100.00

Table 7 presents the descriptive statistics of respondents' satisfaction scores with the overall urban green space in Nanjing. A majority of the respondents, 69.20 % (n = 371), expressed satisfaction (3 and above) with the overall urban green space environment, including those who were basically satisfied (27.80 %, n = 149), satisfied (31.00 %, n = 166), and very satisfied (10.40 %, n = 56). Additionally, the percentages of dissatisfied and very dissatisfied scores were closely aligned, at 15.30 % (n = 82) and 15.50 % (n = 83), respectively.

**Table 7:** Descriptive statistics of urban green space satisfaction.

NO.	Overall satisfaction with urban green space		
	Details	Frequency	(%)
**1**	Very Unsatisfied	82	15.30
**2**	Not satisfied	83	15.50
**3**	Generally satisfied	149	27.80
**4**	satisfied	166	31.00
**5**	Very satisfied	56	10.40
	Total	536	100.00

Moving on to Table 8, it reports the descriptive statistics of respondents' subjective well-being scores. As previously mentioned, happiness assessment scores ranged from 1 to 5, with five indicating the highest level of satisfaction. The results reveal that 77.50 % (n = 415) of the respondents were generally satisfied with their self-reported subjective well-being. These findings support the notion that Nanjing is a city characterized by high levels of happiness, particularly among older adults’ population. Among the respondents, 34.90 % (n = 187) expressed satisfaction and happiness with their current life, while 19.80 % (n = 106) reported feeling very happy.

**Table 8:** Descriptive statistics of subjective well-being scores of older adults.

NO.	subjective well-being scores of older adults		
	Details	Frequency	(%)
**1**	Very unhappy	53	9.90
**2**	Not happy	68	12.70
**3**	General happy	122	22.80
**4**	Happy	187	34.90
**5**	Very happy	106	19.80
	Total	536	100.00

### 3.4 Spatial features of urban green space

Respondents highlighted the importance of their perceptions of different urban green space types in evaluating spatial features related to urban green spaces. All six green space types considered in this study demonstrated significant correlations with satisfaction regarding spatial features: comprehensive parks (p = 0.003), community parks (p = 0.002), wetland parks (p = 0.003), waterfront green spaces (p = 0.006), urban forest parks (p = 0.006), and other types, such as pocket parks (p = 0.035). Interestingly, respondents noted that despite community parks being the most frequently visited type of green space, there was a heightened desire to explore more expansive natural environments and open spaces, particularly due to prolonged movement restrictions.

Among the six listed urban green space types, comprehensive parks (p = 0.012), urban forests (p = 0.008), and wetland parks (p = 0.030) emerged as the primary green space destinations that most frequently attracted older adults to visit and actively participate. The data suggests that the inability of older adults to regularly visit their preferred green space types has a negative impact on their perceptions.

The four major predictors (area, scale, quantity, and quality) associated with the physical space of urban green spaces were deemed very important by the majority of respondents (p < 0.001). The findings underscore those factors such as the availability and accessibility of urban green spaces, distance to parks, and the sense of security were considered crucial by older adults across all discussed categories (p < 0.01), with a particular emphasis on the sense of security (Pearson correlation value of 0.899). Among all the factors analyzed, older adults tended to assign greater importance to factors that might impact safety. This aligns with the results obtained for the grey facilities related to safety in the grey features of urban green spaces mentioned later, such as mobility facility categories (safe paths) and accessibility facilities (ramps). Our findings highlight the significance of the relationship between the spatial features of urban green spaces and the perceptions of green spaces among older adults in the post-epidemic era, with 74.07 % of the indicators passing the significance test.

### 3.5 Green features of urban green space

Our survey focused on determining the green features that are of interest to older adults in urban green spaces. The analysis was based on data obtained from relevant questions in the questionnaire regarding older adults' assessment of green features in these spaces. 11 indicators passed the significance test, accounting for 61.11 %, which means that older people have good results on the perception of green features of urban green spaces in Nanjing. Notably, strong positive correlations were observed in the variables of air purification (p = 0.008), reduction of urban noise (p = 0.003), and vegetation richness (p = 0.005), with two tails. Respondents expressed eagerness to return to nature, breathe fresh air, engage in green sports, and enjoy green landscapes in peaceful urban green spaces once movement restrictions were lifted and city parks were reopened.

Three indicators—ventilation (p = 0.010), purification of soil (p = 0.018), and provision of aesthetic space (p = 0.018)—were highly desirable for older adults in assessing the green features of urban green spaces. Returning to broader green spaces for social interaction allowed them to re-experience the cleanliness, comfort, and aesthetics of these areas, enhancing their overall satisfaction and happiness in life. Additionally, three green features—cooling and humidification (p = 0.020), biodiversity conservation (p = 0.023), and bird and animal species richness (p = 0.023)—were highly valued by respondents during the survey. Conducted in the height of summer, the survey captured the direct experience of older adults seeking shade in tree-covered areas to escape high temperatures and appreciating the biodiversity of green spaces.

Two predictors related to blue space—maintaining carbon and oxygen balance (p = 0.040) and purifying water bodies (p = 0.045)—were associated with indicators of soil conservation and water resources. Most respondents indicated acceptance of the function of urban green spaces in purifying water bodies, aligning with the data processing results.

### 3.6 Grey features of urban green space

The survey focused on grey facilities, four indicators-parking facilities, rest facilities, activity facilities, and sports facilities-displayed significant correlations in both the primary options of frequently used (p-values of 0.048, 0.011, 0.005, and 0.035, respectively) and those needing improvement (p-values 0.022, 0.043, 0.027, 0.024). This underscores that these facilities form the foundation for the physical activities and social interaction of older adults in urban green spaces.

Conversely, the five indicators-sanitation facilities, lighting facilities, safety facilities, management centers, and service facilities—showed opposite trends. The frequently used grey facilities option demonstrated significance for sanitation facilities (p = 0.044), safety facilities (p = 0.015), management centers (p = 0.004), and service facilities (p = 0.006), indicating satisfaction among respondents during use. However, there was no correlation in the grey facilities needing improvement options (p-values of 0.223, 0.113, 0.605, and 0.706, respectively).

The significance of lighting facilities (p = 0.036) in the grey facilities needing improvement option, without correlation in the frequently used grey facilities option, may be attributed to the survey being conducted during clear or cloudy daytime hours, with respondents not utilizing lighting facilities during the survey timeframe.

On the other hand, instructional facilities, landscape facilities, and other facilities did not show significance in the two main options of frequently used (p-values of 0.897, 0.884, and 0.992, respectively) and needing improvement (p-values of 0.602, 0.811, and 0.992, respectively).

In addition, updated deteriorating paths (p = 0.035), enhanced eco-paths (p = 0.033) and accessibility (p = 0.014) were factors of great concern to older adults in this survey. This echoes the spatial safety of urban green spaces mentioned earlier.

### 3.7 Simple linear regression model

Table 9 demonstrates significant correlations between spatial features of urban green spaces (p = 0.007), green features (p = 0.004), and grey features (p = 0.010) and respondents' perceived satisfaction with green spaces. Additionally, Table 10 reveals that respondents' self-reported happiness levels exhibited high correlations with the three main features of urban green spaces, with p-values of 0.029, 0.014, and 0.004, respectively.

**Table 9:** Linear regression results of urban green space features and overall satisfaction with urban green space.

Variables	Overall satisfaction with urban green space						
	95 % CI	Pearson Correlation	*p*-value	Adjusted R-squared	F	t	Durbin-Watson
**Spatial features**	−0.274–0.009	0.117^a^	0.007	0.12	7.450	2.729	1.641
**Green features**	−0.130–0.145	0.125^a^	0.004	0.14	8.430	2.903	1.590
**Grey features**	−0.188–0.084	0.111^a^	0.010	0.10	6.653	2.579	1.603

*Significant at 5 % level.

a Significant at 1% level.

**Table 10:** Linear regression results of urban green space features and subjective well-being of older adults.

Variables	Subjective well-being of older adults						
	95 % CI	Pearson Correlation	*p*-value	Adjusted R-squared	F	t	Durbin-Watson
**Spatial features**	−0.637 ∼ −0.351	0.094^b^	0.029	0.27	4.777	2.186	1.882
**Green features**	−0.493 ∼ −0.216	0.107^a^	0.014	0.10	6.132	2.476	1.922
**Grey features**	−0.549 ∼ −0.279	0.123^a^	0.004	0.13	8.157	2.856	1.874

a Significant at 1% level.

b Significant at 5% level.

The Durbin-Watson values suggest that the observations are mutually independent. However, it is noteworthy that the adjusted R-squared could be enhanced, indicating that the model has relatively poor explanatory power. In an effort to address this, we attempted to construct a regression model using overall satisfaction with green space and the subjective well-being of older adults, as presented in Table 11. Although the p-value of 0.025 indicated a high positive correlation between satisfaction with green space and the subjective well-being of older adults, aligning with research hypothesis 3, it's important to note that the explanatory power of the model still requires improvement (adjusted R-squared of 0.37).

**Table 11:** Linear regression results of overall satisfaction with urban green spaces and subjective well-being of older adults.

Variables	Subjective well-being of older adults						
	95 % CI	Pearson Correlation	*p*-value	Adjusted R-squared	F	t	Durbin-Watson
**Overall satisfaction with urban green space**	−0.501 ∼ −0.223	0.096^a^	0.025	0.37	5.018	2.240	1.893

a Significant at 5% level.

### 3.8 Multiple linear regression model

The preliminary analysis reveals a clear correlation between the indicators within the three main features (study dimensions) of urban green spaces and the satisfaction of older adults with green spaces. While an initial attempt was made to model these data through linear regression for each of the three hypotheses, the models produced were deemed less practically useful. Although most ANOVA tests demonstrated p-values below 0.01, and the generated histograms and standard P–P plots appeared satisfactory, the R-squared and adjusted R-squared fell below 0.5, indicating insufficient explanatory power.

To address this limitation, we optimized the model by employing satisfaction with the three main features of urban green spaces, overall satisfaction with urban green spaces, and the frequency of visits as independent variables. The subjective well-being of older adults was used as the dependent variable for linear regression analysis. This modeling approach was inspired by our research process, where visit frequency represents the degree of older adults' engagement with urban green spaces, satisfaction with the three main features reflects their experiences and perceptions of each feature, and overall satisfaction with urban green spaces captures their overall impression of these spaces in Nanjing.

To test for a linear relationship between the dependent variable and each independent variable, scatter plots were created for each independent variable and the dependent variable separately. The scatter distributions formed approximately straight lines with non-zero slopes, resulting in a useable model with a statistically significant regression equation.

Furthermore, the regression analysis included tests for the normality and homoscedasticity of the residuals, confirming that the residuals exhibited a normal distribution. With an adjusted R-squared of 0.847 (as shown in Table 12), the analysis indicated a high strength of influence among the variables. The Durbin-Watson value of 1.569 suggested that the observations in this multiple linear regression study were mutually independent. The goodness of fit of the regression line to the sample observations was excellent.

**Table 12:** Main parameters of the multiple linear regression model.

Model	R	R-squared	Adjusted R-squared	F	Significance	Durbin-Watson
1	0.921^a^	0.848	0.847	591.459	0.000^b^	1.569

a Dependent variable: subjective well-being of older adults.

b Predictor variables: (constant), satisfaction with spatial features, satisfaction with green features, satisfaction with grey features, overall satisfaction with urban green spaces, frequency of visits.

The ANOVA results displayed an F-value of 591.459, associated with a p-value <0.001, indicating the statistical significance of the regression model at the 0.001 level. This suggests that the effects of the included independent variables on the subjective well-being of older adults were all statistically significant. Consequently, the proposed hypothesis is deemed acceptable. Detailed results are presented in Table 13.

**Table 13:** Regression coefficient and covariance statistics.

Model		Unstandardized coefficient		Standardized coefficient	t	Significance	Covariance statistics	
		B	Standard error	Beta			Tolerance	VIF
1	(Constant)	0.530	0.095		4.727	0.000		
	Spatial feature satisfaction	−0.019	0.016	−0.019	−1.134	0.000	0.973	1.018
	Green feature satisfaction	−0.018	0.017	−0.018	−1.050	0.000	0.972	1.029
	Grey feature satisfaction	−0.025	0.018	−0.024	−1.420	0.000	0.969	1.032
	Overall satisfaction with urban green space	0.913	0.017	0.928	53.214	0.000	0.943	1.061
	Visit frequency	−0.007	0.016	−0.008	−0.463	0.000	0.966	1.035

The ultimate goal of regression analysis is to construct regression models for prediction, and the final multiple linear regression analysis in this study demonstrated the significance of the regression equation.

The regression equation was:

Y = 0.530-0.019 × Spatial feature satisfaction-0.018 × Green feature satisfaction-0.025 × Grey feature satisfaction+0.913 × Overall satisfaction with UGS-0.007 × Visit frequency

## 4 Discussion

### 4.1 Visit frequency, urban green space satisfaction, and subjective well-being of older adults

Our initial hypothesis focuses on establishing a significant correlation between the frequency of visits to greenspaces by older adults and their perceived satisfaction with these spaces, along with its impact on subjective well-being. Our investigation, informed by an extensive literature review and empirical analysis, highlights the frequency of visits as a crucial indicator of older users' engagement with urban green spaces [70]. The participation of older users in these spaces is a structured process known to yield positive outcomes [71] and is fundamental for shaping perceptions of greenspaces [72].

In this survey, the results of the visit frequency of older adults to urban green spaces presented a positive correlation with their subjective well-being, which is in line with the results of previous studies [70,73,74]. Recognizing older adults as a distinct user group in parks and addressing their practical needs, as well as actively encouraging their participation in urban green spaces, can significantly influence the design and management of these areas. Our ongoing research further confirms that maintaining good health is a primary motivation for respondents' visits to urban green spaces. Participants believe that access to and enjoyment of these spaces not only enhances their inclination towards an active lifestyle but also consistently contributes to their overall well-being.

Moreover, access to and engagement with urban green spaces emerges as prerequisites for older adults to fully experience, perceive, and evaluate their satisfaction with the features of such environments. Consequently, the outcomes of our first hypothesis lay the groundwork for exploring potential causal mechanisms, paving the way for subsequent research into our second hypothesis—specifically, the perception of greenspace features.

It is worth noting that the current text doesn't extensively explore disparities in greenspace visitation frequency among older adults based on gender and the subsequent impact on greenspace satisfaction and subjective well-being. This area remains a potential avenue for future research exploration.

### 4.2 Urban green space features on the subjective well-being of older adults

The perception and analysis of older adults’ satisfaction with urban green spaces are challenging because assessing green space perceptions requires understanding the objective features of urban green spaces. Urban green spaces maintain health and enhance well-being by providing an active lifestyle (physical activity) and a vibrant atmosphere [75], which is an indispensable way for older adults to achieve their perception and experience of urban green spaces [76].

#### 4.2.1 The impact of spatial feature of urban green space on the subjective well-being of older adults

We underscore one of the key reasons why spatial features within urban green spaces are pivotal predictors when assessing the subjective well-being of older adults. Throughout the survey, a significant majority of older adults expressed that green spaces, particularly community parks or pocket parks in close proximity to their homes, were their preferred choice for physical exercise or activity-a sentiment consistent with previous studies [77]. These findings indicate that the selection of nearby parks for physical activities may be influenced by factors such as reduced social networks and limited mobility among older adults, potentially influenced by measures implemented for epidemic control. This underscores the considerable reliance of older adults on green spaces for physical activities and symbolically underscores the indispensable role of green spaces in fostering the health and well-being of older populations.

Crucially, our observations indicate that older adults derive social interaction and cultivate enduring friendships through organized social groups, such as dance groups or Tai Chi interest classes, thereby contributing to enhanced subjective well-being. This highlights how urban green spaces serve as the foundational setting for older adults to establish social networks and embody social values [78]. Given the global trend of ageing, our exploration of the spatial features of urban green spaces aims to unravel the intricate relationship between these spaces and the specific green space needs of older adults. Our objective is to create conditions conducive to inclusive urban parks and to facilitate active ageing, aligning with the principles outlined by Yao [79].

In addition, our findings suggest a positive correlation between the impact of the safety of urban green space on older adults' subjective well-being, which coincides with the view that older adults can derive their well-being from the safe urban green space environment [80,81]. Urban green space safety is an essential element in the promotion of social interaction, neighborhood cohesion and subjective well-being among older adults' urban green spaces. Improving barrier-free facilities or building age-friendly roads in the process of renewing urban green space stock not only highlights the significance of the spatial safety of urban green space, but also is an essential embodiment of the core value of age-friendliness in the planning and design plan of urban green space. Age-friendly is a keyword in Nanjing's 2018–2035 urban masterplan, and our study advocates the function of urban green space in maintaining the health and stability of older adults and enhancing their subjective sense of well-being, which is of great implications for both the urban safety pattern and the management of parks in the future.

#### 4.2.2 The impact of green feature of urban green space on the subjective well-being of older adults

In the exploration of the green features within urban green spaces, factors such as noise reduction, vegetation richness, and bird fauna richness emerged as positive and impactful contributors to the healing landscapes experienced through visual, auditory, and tactile sensory levels. This finding aligns with the research conducted by Aw [82] and emphasizes that the green features of urban green spaces influence the well-being of older adults through multiple physiological pathways [83]. Consequently, older adults' perceptions of these green space features play a crucial role in providing an escape from their troubles, aligning with the values of improved health and health maintenance [84].

While the focus of our study predominantly highlights the positive perceptions of older adults regarding the green character of urban green spaces, there is a discernible voice expressing dissatisfaction with monotonous green space landscapes. This dissatisfaction arises from the perceived failure of such landscapes to meet the desire of older adults to actively engage in urban green spaces. This finding not only aligns with the research results of Kaplan [85] and Grahn [86], but also fits in with the critical theoretical achievements of Attention Restoration Theory in the study of urban green spaces and well-being. In assessing the mental health benefits of urban green spaces, particular emphasis was placed on anxiety relief [87], stress reduction [88], attention restoration [89] and enhanced well-being [44]. Notably, older adults experience and perceive urban green space landscapes in a multi-sensory way, which is a holistic process of integrating information from multiple senses. Most of the existing studies focus on the visual impact of urban green space landscapes, while ignoring the impact of other sensory dimensions of urban green space on the physiological health and psychological recovery of older adults. Therefore, government officials or urban landscape designers should fully consider the multi-sensory dimensions of experience and perception of older adults when visiting urban green spaces in order to motivate older adults to actively participate in urban green spaces to achieve more health benefits.

Therefore, our emphasis on the correlation between the green features of urban green spaces and the well-being of older adults serves as a vital avenue for highlighting the positive relationship between the subjective well-being levels of older adults and the natural features of urban green spaces. Our study strongly advocates for active interaction by older adults with the green features of urban green spaces, emphasizing that this engagement contributes to their satisfaction and happiness. It is worth noting that the level of well-being obtained through this pathway is dependent on the level of green features of urban green spaces. In other words, the more pronounced and high-quality the green features of urban green spaces are, the more they induce a high level of subjective well-being in older adults, leading them to perceive green spaces more happily. As such, good-quality urban green spaces are natural landscape configurations that are conducive to survival and wellbeing, can provide high-quality psychological ecosystem services to older adults, reduce stress, alleviate depression, and have a positive impact on health and wellbeing, which coincides with Bratman's [90] direct link between urban green spaces and psychological stress reduction. Our findings can be used as a mediator to understand and address physical and psychological health disparities in older adults, encouraging older adults to engage with urban green spaces and perceive the green features of urban green spaces for more positive emotions, allowing older adults to recover from stressful situations more quickly.

#### 4.2.3 The impact of grey feature of urban green space on the subjective well-being of older adults

Our quantitative study unveils the intricate relationships between perceived grey features within green spaces, corresponding satisfaction levels, and the subjective well-being of older adults. Notably, survey responses highlighted concerns from participants about the lack of convenient grey amenities, such as the absence of public restrooms, worn-out urban furniture, and cluttered plantings in the green spaces surrounding their communities. These deficiencies were identified as deterrents, leading to a reluctance among older adults to visit and engage with these spaces.

Our research underscores those various aspects of grey facilities, including frequently used amenities (11 items), areas in need of improvement (11 items), pathways (4 items), and accessible design, significantly impact older adults' engagement in physical activity, social interaction, as well as their perceptions of safety, walkability, and connectivity within green spaces. These findings align with studies conducted by Lak [91] and Rashidghalam [92].

Accessibility is crucial for older adults to utilize urban green spaces [93], a point that our research substantiates. The planning and design of urban green spaces must consider the affordability of transportation and travel costs for the elderly, which can pose significant challenges [94]. Viewing accessibility as a safe and effective planning tool can not only generate social value but also offer detailed guidance for the revitalization of urban green spaces. By enhancing traditional accessibility measures or creating new age-friendly barrier-free designs, inclusive urban green spaces can be realized. This approach plays a tangible and positive role in facilitating older individuals' access to urban green spaces and fostering their active engagement in various activities within these spaces.

During the survey, elderly park users expressed concerns about the poor quality of paths within and around green spaces, highlighting its significant impact on their engagement in urban green space activities. The importance of creating well-designed and well-connected urban green space transport networks to enhance the health and well-being of residents is emphasized in the United Nations' New Urban Agenda. The focus on accessible quality design and the adaptability of public transportation infrastructure is crucial in addressing spatial inequality and promoting environmental justice, particularly for vulnerable groups like the elderly and disabled individuals [95].

In future urban green space planning, it is essential to accurately identify the needs of the elderly for green space features and promote their active involvement to enhance their satisfaction and well-being. However, in densely populated urban areas with limited space for new green spaces, addressing inequalities in the quantity and quality of urban green spaces requires carefully designed landscapes and facilities tailored to the needs of older adults.

Highlighting the significance of grey features in green spaces for facilitating social interaction is crucial. Easy access to these features supports older adults in engaging in walks, conversations, and outdoor activities, potentially reducing depressive symptoms and anxiety. Additionally, participants emphasized the importance of age-friendly grey facilities and multifunctional spaces for building stronger social relationships and fostering a heightened sense of belonging. Our study further revealed that incorporating age-friendly grey facilities into urban green spaces increased the likelihood of older adults meeting with friends or family, serving as a pivotal avenue for building social networks, fostering a sense of community, and nurturing place attachment. As mentioned earlier, urban green spaces can be seen as an invaluable resource for the provision of psycho-ecological services, and urban green spaces are critical places that can provide not only leisure facilities but also health facilities for older adults. Given what we have found in our study and others, in future research we could try to delve further into the associations between urban green spaces, psychological stress and stress as an effective way to address the negative health outcomes among older adults or different populations. Consequently, the grey infrastructure of urban green spaces plays a vital role in encouraging older adults to visit and form positive perceptions of these areas.

### 4.3 Research contribution

#### 4.3.1 Design perspective

Our research provides insights into the specific features of urban green spaces that significantly impact older adults' perceptions. This knowledge serves as a valuable resource for urban designers and landscape architects seeking practical design breakthroughs. Armed with this information, professionals in these fields can develop more precise measurement tools or design urban green spaces that are not only inclusive but also align with the usage habits and expectations of older adults. This emphasis on age-friendly urban green spaces is crucial for creating environments that cater to the needs of an ageing population.

#### 4.3.2 Policy perspective

Our study offers actionable insights for government departments focused on urban green space planning and the well-being of older adults. It can serve as a policy framework reflecting the preferences of older adults regarding green spaces when formulating local green space management strategies. We advocate for active involvement of older adults in the planning and management of urban green spaces, fostering collaborative efforts with diverse stakeholder groups. This collaborative approach is poised to positively impact the overall maintenance of urban green spaces, enhancing social connectivity, and contributing to the well-being and health of older adults.

This study is appropriate to provide information to Nanjing or cities of the same level about urban green space and the well-being of older adults related to it. In Nanjing's 2018–2035 urban masterplan, green ecology, age-friendly and happy living are highly valued keywords. In the process of new construction, renewal or renovation of urban green spaces, relevant government departments should pay attention to the impact of various features of urban green spaces on their green space satisfaction and subjective well-being. For example, in the process of urban green space renewal, focusing on the richness of vegetation and the quality of health facilities for older adults will contribute to the subjective well-being of older adults.

#### 4.3.3 Theoretical perspective

Through inductive analysis, our research identifies three main features of urban green spaces and distills 3 primary dimensions to establish a correlation with older adults' well-being. We evaluate the subjective well-being of older adults by combining measures of green space satisfaction and visit frequency. This quantitative approach contributes to future theoretical research by providing a nuanced understanding of the relationship between urban green space features and the subjective well-being of older adults. Our findings serve as a foundation for advancing theoretical frameworks in this domain.

In addition, assessing older adults' satisfaction with urban green spaces and their subjective well-being through urban green space features (spatial, green, and grey features), incorporating the theoretical perspectives of Stress Reduction Theory and Attention Restoration Theory, on the one hand, allows our study to explain all the points of discussion from the theoretical point of view. On the other hand, the research findings of this study. On the other hand, the research findings of this study resonate with previous studies and broaden the research horizon to some extent.

## 5 Conclusion

To contribute to the existing knowledge on the relationship between urban green space features and the subjective well-being of older adults, our study was undertaken. In the realm of urban green spaces, the green features hold the most importance for elderly individuals when gauging their satisfaction with these areas. Conversely, when evaluating the subjective well-being of older people, the grey aspects of urban green spaces are crucial. In the post-epidemic era, our findings affirm that a high-quality and secure urban green space environment not only encourages active participation and utilization by older adults but also fosters positive perceptions among them.

Our study is valuable in providing insights into the impact of urban green space features and overall satisfaction with urban green spaces on the subjective well-being of older adults. This study encourages older people's participation in different types of urban green spaces to improve their overall satisfaction with urban green spaces and subjective well-being levels. We also recommend that policy makers, urban planners should pay attention to the importance of the relationship between urban green spaces and older people's subjective well-being in urban master planning, to guide the better incorporation of subjective well-being into new urban development projects that promote older people's health and wellbeing, which is also of great relevance for the development of healthy ageing and happiness cities.

## 6 Limitations and future research

In conclusion, while this research offers distinctive insights, we acknowledge certain limitations that warrant exploration and resolution in future studies. Notably, our study does not delve into the moderating impact of sociodemographic factors among older adults on the relationship between perceived urban green space features and self-reported well-being. Subsequent research endeavors will specifically investigate how sociodemographic features among older adults, coupled with their satisfaction with green spaces, influence the level of subjective well-being. The study collected subjective ratings of urban green space satisfaction and subjective well-being through questionnaires to investigate the influence of different urban green space characteristics on the overall subjective well-being score of elderly individuals. However, the study did not explore how various characteristics of urban green space affect different aspects of subjective well-being (nine indicators in the WOOP scale). Future research should address these gaps to enhance our understanding of this topic, particularly in the context of an increasingly ageing society.

## Ethics statement

Ethical approval and consent to participate.

The authors declare that they have the consent of the Research Ethics Committee of the Universiti Malaya (approval number: M. TNC2/UMREC_2037), and all participants willing agreed to take part in the survey and use their information. All procedures were carried out in compliance with applicable legal and institutional regulations. Adequate protocols were implemented to safeguard the rights and privacy of all participants throughout the study.

## CRediT authorship contribution statement

**Tianrong Xu:** Writing – original draft, Visualization, Validation, Methodology, Investigation, Formal analysis, Data curation, Conceptualization. **Ainoriza Mohd Aini:** Writing – review & editing, Supervision, Conceptualization. **Nikmatul Adha Nordin:** Writing – review & editing, Supervision, Conceptualization.

## Declaration of competing interest

The authors declare that they have no known competing financial interests or personal relationships that could have appeared to influence the work reported in this paper.
