# Supplementary material for: Utilizing regression model to characterize the impact of urban green space features on the subjective well-being of older adults
Source: Heliyon. 2024 Aug 2;10(15):e35567. doi: 10.1016/j.heliyon.2024.e35567 (PMC11336737; doi:10.1016/j.heliyon.2024.e35567)
Supplement: Multimedia component 1 [file mmc1.docx]

Supplementary Material 1: Urban Green Space Sampling

| District | Compreh-  ensive Park | Wetland Park | Waterfront Park | Forest Park | Community Park | Others |
| --- | --- | --- | --- | --- | --- | --- |
|  | **G11** | **EG13** | **EG19** | **EG12** | **G12** | **G139** |
| Gu Lou | Gulou Park | Xindian wetland park | Daqiqo park | Baji hill Park | Xiuqiu Park | Chahaer Lu Pocket Park |
| Qin Huai | Bailuzhou park | Shangqin huai park | Qinhuaihe park | Niushou shan park | Wulongtan Park | Changfu  jie park |
| Xuan Wu | Xuanwu lake park | Huang ma park | Yudaihe park | Jubao shan park | Heping park | Jiangwangmiao park |
| Jian Ye | Mochouhu park | Yuzui wetland park | Binhe park | Youth Olympic Forest Park | Hexi Zhongyang park | Yulanli pocket park |
| Yu Hua Tai | Sanqiao park | Sanqiao wetland park | Xishanqiao waterfront park | Yuhuatai park | Huashenhu park | Zijijhualu pocket park |
| Jiang  Ning | Qilin eco park | Jiulong  hu park | Nanhu park | Fang Shan National Forest Park | Dongshan park | Keyijie pocket park |
| Lu He | Jinniuhu park | Chuhe wan park | Chishanhu park | Pingshan forest park | Taohu park | Siliu pocket park |
| Pu Kou | Pukou park | Pukou jiangtan wetland park | Jiangbian park | Laoshan forest park | Rongzhuang lu park | Qiaolin pocket park |
| Qi Xia | Xianlinhu park | Bagua zhou park | Yaofang men park | Qixiashan park | Happiness river park | Sanyuan jiayuan park |
| Li Shui | Qin huai yuan park | Xing zhuang wetland park | Yulu lake park | Wu xiang shan forest park | Zhuang yuan  Fang culture park | Nan yuan pocket park |
| Gao Chun | Baota park | Guchen hu park | Laizhuzhou park | Youzi shan park | Panchi Park | Qiqiao park |

**Supplementary Material 2: Collinearity Diagnostics for Independent Variables**

| NO. | Variables | VIF | Tolerance | Conditional indicators |
| --- | --- | --- | --- | --- |
| 1 | Spatial features | 1.04 | 0.96 | 10.66 |
| 2 | Green features | 1.04 | 0.95 | 11.34 |
| 3 | Gray features | 1.05 | 0.94 | 16.97 |
| 4 | Overall satisfaction with urban green space | 1.89 | 0.52 | 28.54 |
| 5 | Visit frequency | 1.03 | 0.96 | 7.82 |
